# Supplementary material for: Modeling glioblastoma heterogeneity as a dynamic network of cell states
Source: Mol Syst Biol. 2021 Sep 16;17(9):e10105. doi: 10.15252/msb.202010105 (PMC8444284; doi:10.15252/msb.202010105)
Supplement: Supplementary file 5 — Source Data for Figure 3 [file MSB-17-e10105-s001.zip › Figure3A_sourcedata/GSEA_3065/hallmarks_state3.GseaPreranked.1623416274744/HALLMARK_E2F_TARGETS.html]

Details for gene set HALLMARK\_E2F\_TARGETS[GSEA]

|  || Dataset | state3 |
| Phenotype | NoPhenotypeAvailable |
| Upregulated in class | na\_pos |
| GeneSet | HALLMARK\_E2F\_TARGETS |
| Enrichment Score (ES) | 0.3093949 |
| Normalized Enrichment Score (NES) | 1.3197548 |
| Nominal p-value | 0.02846975 |
| FDR q-value | 0.1475778 |
| FWER p-Value | 0.644 |
Table: GSEA Results Summary

  

Fig 1: Enrichment plot: HALLMARK\_E2F\_TARGETS      
 Profile of the Running ES Score & Positions of GeneSet Members on the Rank Ordered List

  

| PROBE | GENE SYMBOL | GENE\_TITLE | RANK IN GENE LIST | RANK METRIC SCORE | RUNNING ES | CORE ENRICHMENT || 1 | PRKDC |  |  | 6 | 0.588 | 0.0269 | Yes |
| 2 | PRDX4 |  |  | 41 | 0.455 | 0.0442 | Yes |
| 3 | TOP2A |  |  | 42 | 0.448 | 0.0653 | Yes |
| 4 | MKI67 |  |  | 59 | 0.407 | 0.0825 | Yes |
| 5 | NASP |  |  | 85 | 0.360 | 0.0964 | Yes |
| 6 | CENPE |  |  | 126 | 0.321 | 0.1068 | Yes |
| 7 | LBR |  |  | 131 | 0.319 | 0.1213 | Yes |
| 8 | DNMT1 |  |  | 149 | 0.310 | 0.1338 | Yes |
| 9 | KPNA2 |  |  | 157 | 0.308 | 0.1474 | Yes |
| 10 | DLGAP5 |  |  | 207 | 0.279 | 0.1547 | Yes |
| 11 | HELLS |  |  | 218 | 0.275 | 0.1665 | Yes |
| 12 | SMC1A |  |  | 231 | 0.271 | 0.1778 | Yes |
| 13 | PNN |  |  | 256 | 0.260 | 0.1872 | Yes |
| 14 | SMC4 |  |  | 265 | 0.255 | 0.1982 | Yes |
| 15 | ATAD2 |  |  | 282 | 0.249 | 0.2080 | Yes |
| 16 | LUC7L3 |  |  | 289 | 0.247 | 0.2189 | Yes |
| 17 | BRCA1 |  |  | 339 | 0.232 | 0.2241 | Yes |
| 18 | SMC3 |  |  | 341 | 0.231 | 0.2348 | Yes |
| 19 | NUP107 |  |  | 364 | 0.227 | 0.2428 | Yes |
| 20 | XPO1 |  |  | 390 | 0.220 | 0.2503 | Yes |
| 21 | TMPO |  |  | 420 | 0.214 | 0.2569 | Yes |
| 22 | HMMR |  |  | 478 | 0.202 | 0.2597 | Yes |
| 23 | MCM4 |  |  | 482 | 0.201 | 0.2688 | Yes |
| 24 | MMS22L |  |  | 645 | 0.179 | 0.2583 | Yes |
| 25 | CSE1L |  |  | 652 | 0.178 | 0.2659 | Yes |
| 26 | LIG1 |  |  | 691 | 0.171 | 0.2695 | Yes |
| 27 | MRE11 |  |  | 822 | 0.155 | 0.2616 | Yes |
| 28 | NOP56 |  |  | 829 | 0.155 | 0.2682 | Yes |
| 29 | MAD2L1 |  |  | 846 | 0.153 | 0.2735 | Yes |
| 30 | BUB1B |  |  | 857 | 0.152 | 0.2794 | Yes |
| 31 | MSH2 |  |  | 885 | 0.150 | 0.2833 | Yes |
| 32 | CDK1 |  |  | 915 | 0.147 | 0.2868 | Yes |
| 33 | MCM7 |  |  | 949 | 0.143 | 0.2896 | Yes |
| 34 | ORC6 |  |  | 1000 | 0.138 | 0.2903 | Yes |
| 35 | LMNB1 |  |  | 1021 | 0.137 | 0.2944 | Yes |
| 36 | IPO7 |  |  | 1098 | 0.130 | 0.2916 | Yes |
| 37 | GINS4 |  |  | 1104 | 0.129 | 0.2970 | Yes |
| 38 | BRCA2 |  |  | 1122 | 0.128 | 0.3010 | Yes |
| 39 | ILF3 |  |  | 1176 | 0.123 | 0.3006 | Yes |
| 40 | RAD51AP1 |  |  | 1196 | 0.122 | 0.3041 | Yes |
| 41 | TFRC |  |  | 1304 | 0.115 | 0.2970 | Yes |
| 42 | POLE |  |  | 1305 | 0.115 | 0.3024 | Yes |
| 43 | GSPT1 |  |  | 1324 | 0.114 | 0.3057 | Yes |
| 44 | SPAG5 |  |  | 1357 | 0.111 | 0.3071 | Yes |
| 45 | NUP205 |  |  | 1397 | 0.108 | 0.3077 | Yes |
| 46 | CIT |  |  | 1426 | 0.107 | 0.3094 | Yes |
| 47 | ANP32E |  |  | 1548 | 0.101 | 0.3000 | No |
| 48 | SRSF2 |  |  | 1697 | 0.092 | 0.2870 | No |
| 49 | MCM2 |  |  | 1762 | 0.088 | 0.2837 | No |
| 50 | WDR90 |  |  | 1764 | 0.088 | 0.2877 | No |
| 51 | KIF18B |  |  | 1816 | 0.086 | 0.2858 | No |
| 52 | AURKB |  |  | 1838 | 0.084 | 0.2873 | No |
| 53 | MCM3 |  |  | 1918 | 0.080 | 0.2818 | No |
| 54 | PAICS |  |  | 1931 | 0.079 | 0.2841 | No |
| 55 | EXOSC8 |  |  | 1955 | 0.078 | 0.2851 | No |
| 56 | KIF2C |  |  | 1985 | 0.077 | 0.2853 | No |
| 57 | CBX5 |  |  | 2001 | 0.076 | 0.2871 | No |
| 58 | MXD3 |  |  | 2058 | 0.074 | 0.2840 | No |
| 59 | STAG1 |  |  | 2064 | 0.073 | 0.2868 | No |
| 60 | RAD21 |  |  | 2150 | 0.070 | 0.2802 | No |
| 61 | POLD3 |  |  | 2204 | 0.068 | 0.2772 | No |
| 62 | CDCA3 |  |  | 2232 | 0.067 | 0.2772 | No |
| 63 | MCM6 |  |  | 2237 | 0.067 | 0.2799 | No |
| 64 | DEPDC1 |  |  | 2259 | 0.066 | 0.2805 | No |
| 65 | EED |  |  | 2266 | 0.066 | 0.2829 | No |
| 66 | NUP153 |  |  | 2274 | 0.065 | 0.2851 | No |
| 67 | CDCA8 |  |  | 2281 | 0.065 | 0.2874 | No |
| 68 | MYC |  |  | 2324 | 0.064 | 0.2855 | No |
| 69 | PDS5B |  |  | 2342 | 0.063 | 0.2865 | No |
| 70 | MCM5 |  |  | 2344 | 0.063 | 0.2893 | No |
| 71 | RFC1 |  |  | 2360 | 0.062 | 0.2905 | No |
| 72 | MLH1 |  |  | 2369 | 0.062 | 0.2925 | No |
| 73 | SRSF1 |  |  | 2450 | 0.059 | 0.2859 | No |
| 74 | CHEK1 |  |  | 2475 | 0.058 | 0.2858 | No |
| 75 | DIAPH3 |  |  | 2493 | 0.057 | 0.2865 | No |
| 76 | BARD1 |  |  | 2514 | 0.056 | 0.2868 | No |
| 77 | KIF22 |  |  | 2545 | 0.055 | 0.2859 | No |
| 78 | UBE2T |  |  | 2599 | 0.054 | 0.2822 | No |
| 79 | ZW10 |  |  | 2625 | 0.052 | 0.2818 | No |
| 80 | XRCC6 |  |  | 2637 | 0.052 | 0.2829 | No |
| 81 | WEE1 |  |  | 2687 | 0.050 | 0.2796 | No |
| 82 | RFC3 |  |  | 2708 | 0.050 | 0.2796 | No |
| 83 | RACGAP1 |  |  | 2769 | 0.048 | 0.2748 | No |
| 84 | CTPS1 |  |  | 2789 | 0.047 | 0.2748 | No |
| 85 | RRM2 |  |  | 2835 | 0.046 | 0.2717 | No |
| 86 | GINS1 |  |  | 2840 | 0.046 | 0.2734 | No |
| 87 | NBN |  |  | 3008 | 0.041 | 0.2558 | No |
| 88 | ING3 |  |  | 3019 | 0.041 | 0.2565 | No |
| 89 | EIF2S1 |  |  | 3099 | 0.038 | 0.2491 | No |
| 90 | DONSON |  |  | 3191 | 0.036 | 0.2402 | No |
| 91 | KIF4A |  |  | 3195 | 0.036 | 0.2415 | No |
| 92 | CCP110 |  |  | 3323 | 0.033 | 0.2282 | No |
| 93 | TIPIN |  |  | 3336 | 0.032 | 0.2283 | No |
| 94 | NCAPD2 |  |  | 3371 | 0.031 | 0.2258 | No |
| 95 | SPC24 |  |  | 3409 | 0.030 | 0.2229 | No |
| 96 | RBBP7 |  |  | 3423 | 0.030 | 0.2228 | No |
| 97 | MELK |  |  | 3462 | 0.029 | 0.2197 | No |
| 98 | TIMELESS |  |  | 3497 | 0.028 | 0.2171 | No |
| 99 | CCNB2 |  |  | 3502 | 0.028 | 0.2179 | No |
| 100 | AURKA |  |  | 3503 | 0.028 | 0.2192 | No |
| 101 | CNOT9 |  |  | 3541 | 0.027 | 0.2162 | No |
| 102 | SMC6 |  |  | 3571 | 0.026 | 0.2140 | No |
| 103 | SPC25 |  |  | 3575 | 0.026 | 0.2149 | No |
| 104 | PRPS1 |  |  | 3706 | 0.022 | 0.2008 | No |
| 105 | POLA2 |  |  | 3855 | 0.019 | 0.1844 | No |
| 106 | PCNA |  |  | 3986 | 0.016 | 0.1699 | No |
| 107 | CDC25A |  |  | 4065 | 0.013 | 0.1614 | No |
| 108 | TRIP13 |  |  | 4114 | 0.012 | 0.1564 | No |
| 109 | PRIM2 |  |  | 4130 | 0.012 | 0.1552 | No |
| 110 | TACC3 |  |  | 4146 | 0.011 | 0.1540 | No |
| 111 | PSIP1 |  |  | 4250 | 0.008 | 0.1423 | No |
| 112 | ORC2 |  |  | 4299 | 0.007 | 0.1370 | No |
| 113 | PA2G4 |  |  | 4363 | 0.005 | 0.1299 | No |
| 114 | HMGB3 |  |  | 4445 | 0.003 | 0.1206 | No |
| 115 | DDX39A |  |  | 4706 | -0.004 | 0.0904 | No |
| 116 | SYNCRIP |  |  | 4713 | -0.004 | 0.0899 | No |
| 117 | PMS2 |  |  | 4727 | -0.004 | 0.0886 | No |
| 118 | EZH2 |  |  | 4952 | -0.010 | 0.0629 | No |
| 119 | DCK |  |  | 4959 | -0.010 | 0.0627 | No |
| 120 | UNG |  |  | 4979 | -0.010 | 0.0609 | No |
| 121 | ASF1B |  |  | 5007 | -0.011 | 0.0583 | No |
| 122 | RAD1 |  |  | 5054 | -0.012 | 0.0535 | No |
| 123 | RPA2 |  |  | 5078 | -0.013 | 0.0514 | No |
| 124 | UBR7 |  |  | 5204 | -0.016 | 0.0376 | No |
| 125 | PLK1 |  |  | 5235 | -0.017 | 0.0348 | No |
| 126 | PPP1R8 |  |  | 5323 | -0.019 | 0.0256 | No |
| 127 | LYAR |  |  | 5437 | -0.022 | 0.0134 | No |
| 128 | HMGB2 |  |  | 5479 | -0.023 | 0.0097 | No |
| 129 | DSCC1 |  |  | 5503 | -0.024 | 0.0082 | No |
| 130 | SLBP |  |  | 5661 | -0.029 | -0.0088 | No |
| 131 | TP53 |  |  | 5700 | -0.030 | -0.0118 | No |
| 132 | CCNE1 |  |  | 5730 | -0.031 | -0.0138 | No |
| 133 | CTCF |  |  | 5766 | -0.032 | -0.0164 | No |
| 134 | CDKN1B |  |  | 5783 | -0.032 | -0.0167 | No |
| 135 | CDC20 |  |  | 5818 | -0.033 | -0.0191 | No |
| 136 | CENPM |  |  | 5855 | -0.034 | -0.0217 | No |
| 137 | NUDT21 |  |  | 5874 | -0.035 | -0.0222 | No |
| 138 | USP1 |  |  | 5963 | -0.038 | -0.0307 | No |
| 139 | PSMC3IP |  |  | 6105 | -0.043 | -0.0451 | No |
| 140 | RPA1 |  |  | 6115 | -0.044 | -0.0441 | No |
| 141 | PPM1D |  |  | 6214 | -0.047 | -0.0534 | No |
| 142 | NOLC1 |  |  | 6241 | -0.048 | -0.0542 | No |
| 143 | SHMT1 |  |  | 6445 | -0.054 | -0.0753 | No |
| 144 | TRA2B |  |  | 6483 | -0.056 | -0.0770 | No |
| 145 | CKS2 |  |  | 6569 | -0.059 | -0.0842 | No |
| 146 | TCF19 |  |  | 6657 | -0.064 | -0.0913 | No |
| 147 | HNRNPD |  |  | 6663 | -0.064 | -0.0889 | No |
| 148 | MTHFD2 |  |  | 6745 | -0.068 | -0.0952 | No |
| 149 | PHF5A |  |  | 6795 | -0.071 | -0.0976 | No |
| 150 | ASF1A |  |  | 6818 | -0.072 | -0.0968 | No |
| 151 | HUS1 |  |  | 6879 | -0.075 | -0.1003 | No |
| 152 | CDC25B |  |  | 6935 | -0.078 | -0.1031 | No |
| 153 | TBRG4 |  |  | 7225 | -0.095 | -0.1323 | No |
| 154 | RAD51C |  |  | 7264 | -0.098 | -0.1322 | No |
| 155 | SSRP1 |  |  | 7499 | -0.117 | -0.1540 | No |
| 156 | NAP1L1 |  |  | 7538 | -0.121 | -0.1528 | No |
| 157 | TK1 |  |  | 7568 | -0.124 | -0.1504 | No |
| 158 | CKS1B |  |  | 7569 | -0.124 | -0.1446 | No |
| 159 | DUT |  |  | 7608 | -0.128 | -0.1430 | No |
| 160 | CDKN2C |  |  | 7609 | -0.128 | -0.1370 | No |
| 161 | SNRPB |  |  | 7671 | -0.136 | -0.1377 | No |
| 162 | BIRC5 |  |  | 7735 | -0.144 | -0.1383 | No |
| 163 | RFC2 |  |  | 7739 | -0.144 | -0.1319 | No |
| 164 | MYBL2 |  |  | 7771 | -0.148 | -0.1286 | No |
| 165 | STMN1 |  |  | 7808 | -0.153 | -0.1256 | No |
| 166 | NME1 |  |  | 7811 | -0.154 | -0.1186 | No |
| 167 | TUBB |  |  | 7813 | -0.154 | -0.1116 | No |
| 168 | DCTPP1 |  |  | 7845 | -0.158 | -0.1078 | No |
| 169 | TUBG1 |  |  | 7966 | -0.179 | -0.1134 | No |
| 170 | RAN |  |  | 7979 | -0.181 | -0.1063 | No |
| 171 | CDKN3 |  |  | 7998 | -0.184 | -0.0997 | No |
| 172 | RPA3 |  |  | 8014 | -0.188 | -0.0927 | No |
| 173 | AK2 |  |  | 8036 | -0.191 | -0.0862 | No |
| 174 | NAA38 |  |  | 8114 | -0.206 | -0.0855 | No |
| 175 | RANBP1 |  |  | 8132 | -0.212 | -0.0776 | No |
| 176 | CDK4 |  |  | 8168 | -0.220 | -0.0713 | No |
| 177 | POLE4 |  |  | 8169 | -0.220 | -0.0610 | No |
| 178 | JPT1 |  |  | 8212 | -0.231 | -0.0551 | No |
| 179 | PTTG1 |  |  | 8366 | -0.279 | -0.0599 | No |
| 180 | DEK |  |  | 8376 | -0.284 | -0.0476 | No |
| 181 | POP7 |  |  | 8436 | -0.308 | -0.0400 | No |
| 182 | HMGA1 |  |  | 8519 | -0.346 | -0.0334 | No |
| 183 | POLD2 |  |  | 8549 | -0.363 | -0.0197 | No |
| 184 | UBE2S |  |  | 8655 | -0.458 | -0.0105 | No |
| 185 | CDKN1A |  |  | 8657 | -0.461 | 0.0110 | No |
Table: GSEA details [plain text format]

  

Fig 2: HALLMARK\_E2F\_TARGETS: Random ES distribution      
 Gene set null distribution of ES for **HALLMARK\_E2F\_TARGETS**
